# Establishment of a simple prediction method for DNA melting temperature: high-resolution melting curve analysis of PCR products

**DOI:** 10.1371/journal.pone.0321885

**Published:** 2025-04-16

**Authors:** Yunchao Zhou, Shan Ha, Yuzhao Xu, Xiaoshi Qin, Yixin Ma, Jianghuan Lu, Bo Wang, Jie Cai, Zhiao Duan, Bin Cong, Jianhua Chen, Jianqiang Deng

**Affiliations:** 1 Hainan Provincial Tropical Forensic Engineering Research Center & Hainan Provincial Academician Workstation (Tropical Forensic Medicine), Key Laboratory of Tropical Translational Medicine of Ministry of Education, School of Basic Medicine and Life Sciences, Hainan Medical University, Haikou, China; 2 Hainan Vocational College of Political Science and Law, Haikou, China; 3 Department of Forensic Medicine, Hebei Medical University, Shijiazhuang, China; Bristol-Myers Squibb Company, UNITED STATES OF AMERICA

## Abstract

High-resolution melting analysis is a technique that leverages the principle that the thermal stability of dsDNA is influenced by its length and base composition. This method generates a melting curve by real-time monitoring of the changes in fluorescence signal as dsDNA melts during the heating process. The melting temperature serves as a fundamental indicator of sample characteristics in HRM analysis. During the initial stages of designing a new HRM experimental system, accurately predicting the Tm position of the established system can significantly enhance research efficiency. Currently, there is a limited number of studies focused on the prediction of Tm values in HRM analysis, with varying levels of predictive accuracy. The nearest-neighbor method model can well reflect the interaction of adjacent base pairs. Therefore, we combined the nearest neighbor method model and applied parameters such as enthalpy change, entropy change, GC content and number of base pairs of the DNA sequence to derive a new empirical formula for predicting Tm values. In this study, five species of seawater diatoms were selected as the research subjects. Four specific primers were employed to amplify the extracted DNA through PCR, and the resulting amplified products underwent HRM analysis and Sanger sequencing. Based on the obtained DNA sequences, we calculated the corresponding GC content, number of base pairs, enthalpy change and entropy change, combined with the Tm value obtained from the experiment. Finally, the following formula for predicting Tm value is obtained: (1) When the GC content is 40%≤GC content≤60%: Tm=ΔH/ΔS–0.27GC%–(150+2n)/n–273.15; (2) When the GC content is <40%: Tm=ΔH/ΔS–GC%/3–(150+2n)/n–273.15. After that, the DNA sequences amplified using two other specific primers were verified, and the predicted Tm values were compared with the measured values. The average error was within 1 °C.The results show that the formula obtained in the study can accurately predict the Tm value, which can be effectively used to identify the species of unknown samples.Therefore, this Tm value prediction method provides new methods and ideas for solving practical problems in multiple related fields.

## Introduction

High-resolution melting (HRM) analysis is an emerging technology based on Post-Polymerase Chain Reaction (post-PCR) analysis, which is mainly used to identify differences in nucleic acid sequences [1,2]. This technology is simple, efficient, and cost-effective, allowing for quick genotyping of known mutations or scanning for unknown ones. It’s suitable for fields like biomedical diagnostics, forensic species research, wildlife conservation, and entomological classification [3–6]. This technology takes advantage of the thermal stability of double-stranded DNA(dsDNA), which is affected by its length and base composition. It generates melting curves by monitoring in real time the change in fluorescence signals during the melting of dsDNA in the process of temperature rise [7]. Since sequence variations can lead to changes in the melting behavior of dsDNA during temperature rise, minor differences in melting curves can be used to detect sequence variations in PCR fragments [8].

Melting temperature (Tm) is the temperature at which half of the double helix is broken down into single strands during the thermal denaturation of DNA. The temperature is mainly determined by the length of the sequence and Guanine-Cytosine (GC) content. In HRM, it is expressed by the horizontal coordinate corresponding to the peak value of the first derivative curve (melting curve), which represents the core test index of sample characteristics [8,9]. At the beginning of designing a new HRM experimental system, if we can accurately predict the Tm position of the established system, it will help to improve the efficiency of the study. In particular, research is currently beginning to focus on realizing HRM’s inspection capabilities for mixed samples through multiplex high-resolution melt-curve analysis [10,11]. The key is to distribute the Tm values of different species obtained by the established HRM system at different temperature positions to achieve differentiation. If a prediction method for Tm values in HRM technology is established, there is no need to first verify its usability through actual experiments for each newly established solution to achieve effective preliminary screening, saving a lot of manpower. Therefore, establishing a simple, fast, and relatively accurate prediction method for Tm value in HRM technology is of great value in the construction of HRM technology.

Currently, there’s not much research on predicting Tm values in HRM. The few methods available, whether they rely solely on DNA sequence composition or are based on salinity correction, all show pretty poor accuracy in their predictions [12,13]. The nearest-neighbor method is a relatively good method in terms of accuracy of prediction results [14,15]. However, some parameters involved in its formula are difficult to obtain, and the formula is complex and difficult to master. These limitations make it difficult to apply this method in many grassroots laboratories. In addition, these methods are usually only applicable to DNA sequences with a length of 16~30 bp and cannot effectively predict the Tm value of longer DNA sequences. It has limited value in the practical application of HRM. Given this, this study intends to use diatoms as the research object to explore a more efficient and simpler method for predicting Tm values in HRM analysis.

We performed melting experiments on PCR products to obtain the true Tm values of different products. This is to better reflect the actual situation of performing melting analysis directly after PCR amplification. The main factors affecting Tm value include the sequence of base pairs, DNA length, and GC content. Therefore, based on the DNA sequences of 28 PCR products obtained, we proposed a new empirical formula to predict Tm values in HRM analysis using the nearest neighbor thermodynamic model, and validated it. This method demonstrates high prediction accuracy and practicality.

## Materials and methods

### Experimental samples

In this study, we used five species of pure diatoms: *Nitzschia sp*. (MASCC-0037), *Odontella sp.* (MASCC-0019), *Skeletonema sp.* (MASCC-0022), *Chaetoceros sp.* (MASCC-0031), and *Navicula sp.* (MASCC-0035). We purchased them from the Seaweed Culture Collection Center, Institute of Oceanology, Chinese Academy of Sciences. We cultured them in Enriched Seawater Medium “f/2” at a temperature of 21 °C, with lighting set to 1500 lux for 12 hours of light and 12 hours of dark.

### DNA extraction from diatom

Diatom cells have a special structure with a hard siliceous shell. This study adopted an improved diatom DNA extraction method designed and improved by our laboratory in previous research. The specific method can be found in Reference [16].

### PCR and obtaining HRM measured values

Determine the concentration of extracted DNA. we selected 6 pairs of diatom-specific primers (*UPA99* [17], *18S-V7* [18], *ND* [19], *Primer1* [20], *psaA-2* [21], and *Dia18S*) for PCR amplification. The primer sequences are shown in Table 1, and the amplification system is shown in Table 2. The PCR reaction conditions of primer *UPA99* are shown in Table 3. The PCR reaction conditions of primers *18S-V7*, *ND*, *Primer1*, *psaA-2* and *Dia18S* are shown in Table 4. The PCR amplification products were subjected to HRM analysis on the Rotor-GeneQ real-time fluorescence quantitative PCR instrument. The analysis conditions were as follows: full denaturation at 95 °C for 1 min; gradual heating from 60 °C to 95 °C, ramp rate 0.1 °C/s. Thus, the measured Tm values of different diatoms and different primers were obtained.

**Table 1:** Information on the specific primers used for diatoms.

Primer Name	Primer Sequence 5’ to 3’	Annealing Temperature
*UPA99-F*	GGAAACGTACAAAGGTTTCC	59 °C
*UPA99-R*	GTCGCTGCACGACTTGTAGG	
*18S-V7F*	TCCGTTAACGAACGAGACC	58 °C
*18S-V7R*	GGCATCACAGACCTGTTATTG	
*ND-F*	ATTAGGTTTATCTGGTAAAAACTAC	51 °C
*ND-R*	TTCTCTCCAACGCATGAA	
*Primer1-F*	CAATTGGAGGGCAAGTCTGG	58 °C
*Primer1-R*	CCTGCACGACAACCTAATG	
*psaA-2-F*	GGCACTGGATCTACACGGTA	61 °C
*psaA-2-R*	TCCGTATCACACCACAACCA	
*Dia18S-F*	AGTTGTTGCAGTNAAAAGCTCG	53 °C
*Dia18S-R*	GTAAANGATGGGCATCCCCT	

**Table 2:** PCR system.

Reagents	Dosage
template DNA	4pg–8ng
MonAmp^TM^ 2×Taq mix Pro	10μL
Primer F (10μM)	1μL
Primer R (10μM)	1μL
20×Eva Green	1μL
ddH_2_O	to 20 µL
Total	20μL

**Table 3:** PCR condition 1.

Step	Procedure	Temperature	Times	
1	Initial Denaturation	95 °C	2min	
2	Denaturation	94 °C	30s	35cycles
	Annealing	59 °C	60s	
	Extension	72 °C	60s	
3	Final Extension	72 °C	10min	

**Suitable for amplification of primers *UPA99***.

**Table 4:** PCR condition 2.

Step	Procedure	Temperature	Times	
1	Initial Denaturation	95 °C	10min	
2	Denaturation	94 °C	45s	35cycles
	Annealing	X	45s	
	Extension	72 °C	60s	
3	Final Extension	72 °C	10min	

**Suitable for amplification of primers *18S-V7*, *ND*, *Primer1*, *psaA-2, Dia18S*.**

**The corresponding temperature of annealing temperature X is shown in Table 1**

### DNA sequencing and target sequence determination

The PCR amplification products after HRM analysis will not be damaged and can be subjected to relevant subsequent analysis. Therefore, the amplification products of each primer were directly sent to Taihe Biotechnology (Beijing) Co., Ltd. for Sanger sequencing. The obtained sequences were compared using the NCBI database to verify whether the primer PCR amplification product was the sequence of the target algal species.

### Establishment of Tm value prediction model in HRM

Organized the DNA sequences obtained through sequencing. Used the nearest neighbor method proposed by Allawi and SantLucia(1997 and 1998) [15,22] with thermodynamic parameters of (Table 5) to calculate the enthalpy change (ΔH) and entropy change (ΔS) for different DNA sequences, as well as to determine the GC content and number of base pairs for each sequence. Established the Tm prediction formula using the DNA sequences amplified by the four pairs of primers: *UPA99-F*/*UPA99-R*, *18S-V7F*/*18S-V7R*, *ND-F*/ND-R, and *Primer1-F*/*Primer1-R*.

**Table 5:** Thermodynamic parameters of the nearest neighbor method proposed by Allawi and SantLucia (1997 and 1998) [15,22].

Sequence	ΔH° (kcal/mol)	ΔS° (cal/k·mol)
AA/TT	−7.9	−22.2
AT/TA	−7.2	−20.4
TA/AT	−7.2	−21.3
CA/GT	−8.5	−22.7
GT/CA	−8.4	−22.4
CT/GA	−7.8	−21.0
GA/CT	−8.2	−22.2
CG/GC	−10.6	−27.2
GC/CG	−9.8	−24.4
GG/CC	−8.0	−19.9
Init. w/term. G·C	0.1	−2.8
Init. w/term. A·T	2.3	4.1
Symmetry correction	0	−1.4

Taking a short nucleotide sequence GTGCAT/ATGCAC as an example, we used the thermodynamic parameters from Table 5 to demonstrate the specific calculation process for ΔH and ΔS:


$$\begin{array}{l} \Delta H=\Delta H{}^{\circ}\left(GT/CA\right)+\Delta H{}^{\circ}\left(TG/AC\right)+\Delta H{}^{\circ}\left(GC/CG\right)+\Delta H{}^{\circ}\left(CA/GT\right)+\Delta H{}^{\circ}\left(AT/TA\right)+\\ \Delta H{}^{\circ}\left(InitG\cdot C\right)+\Delta H{}^{\circ}\left(InitA\cdot T\right)+\Delta H{}^{\circ}\left(Sym\right)=-8.4－8.5－9.8－8.5－7.2+0.1+2.3+0=-40\\ kcal/mol \end{array}$$

$$\begin{array}{l} \Delta S=\Delta S{}^{\circ}\left(GT/CA\right)+\Delta S{}^{\circ}\left(TG/AC\right)+\Delta S{}^{\circ}\left(GC/CG\right)+\Delta S{}^{\circ}\left(CA/GT\right)+\Delta S{}^{\circ}\left(AT/TA\right)+\Delta S{}^{\circ}\\ \left(InitG\cdot C\right)+\Delta S{}^{\circ}\left(InitA\cdot T\right)+\Delta S{}^{\circ}\left(Sym\right)=-22.4－22.7－24.4－22.7－20.4－2.8+4.1－1.4=-112.7\\ cal/k\cdot mol \end{array}$$

### Validation of established predictive models

After establishing the formula, the two pairs of primers *psaA-2-F*/*psaA-2-R* and *Dia18S-F*/*Dia18S-R* and their products were used for verification. The predicted values were obtained through the prediction model, and the actual values were obtained through actual experiments. The error between the actual and predicted values was analyzed.

## Results

### HRM analysis results

**Fig 1:**
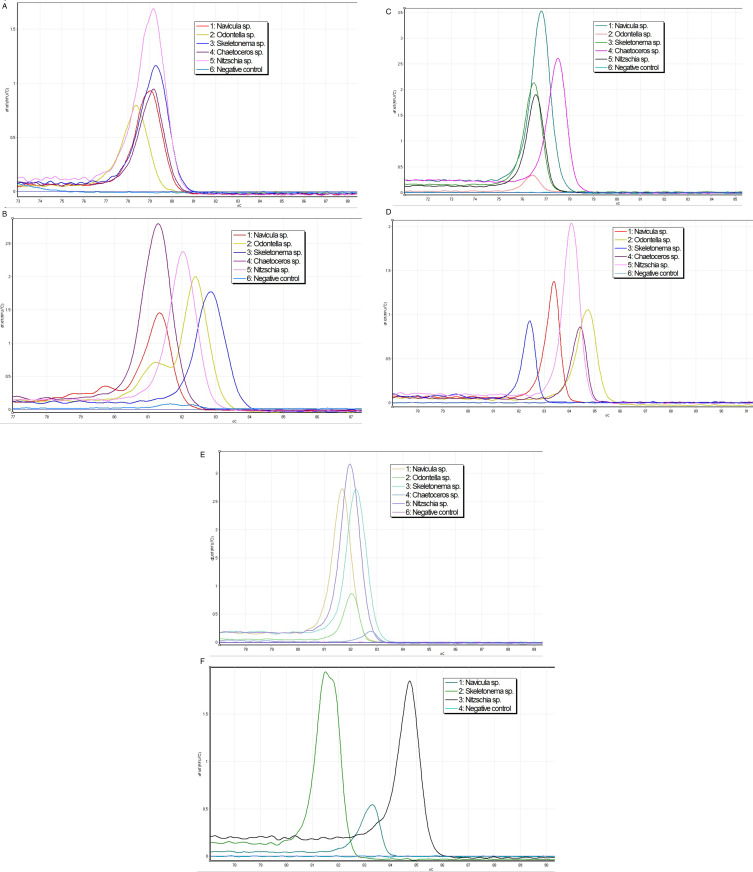
Melting curves obtained after PCR amplification of five different species of marine diatoms using four different diatom-specific primers. (A) shows the melting curves obtained after PCR amplification of different diatoms using primer *UPA99-F*/*UPA99-R*; (B) shows the melting curves obtained after PCR amplification of different diatoms using primer *18S-V7F*/*18S-V7R*; (C) shows the melting curves obtained after PCR amplification of different diatoms using primer *ND-F*/ND-R;(D)shows the melting curves obtained after PCR amplification of different diatoms using primer *Primer1-F*/*Primer1-R*; (E) shows the melting curves obtained after PCR amplification of different diatoms using primer *psaA-2-F*/*psaA-2-R*; (F) shows the melting curves obtained after PCR amplification of different diatoms using primer *Dia18S-F*/*Dia18S-R*.

Primers *UPA99-F*/*UPA99-R*, *18S-V7F*/*18S-V7R*, *ND-F*/ND-R, *Primer1-F*/*Primer1-R*, and *psaA-2-F*/*psaA-2-R* all specifically amplified five species of marine diatoms, and each primer yielded five distinct melting curves (derivative curves) after HRM analysis. Primer *Dia18S-F*/*Dia18S-R* could only specifically amplify *Nitzschia sp., Skeletonema sp., and Navicula sp.*, resulting in only three distinct melting curves after HRM analysis. Different diatoms have different Tm values and unique melting curves, and no detection peaks were observed in the negative control group (Fig 1). The results indicate that different species can be distinguished by different Tm values and melting curve shapes.

### Measured Tm values before establishing the predictive model

Each curve forms a specific detection peak, with the x-axis representing temperature. The temperature corresponding to the peak value is the melting temperature of the DNA amplification product. Using 4 specific primers, we carried out PCR amplification on 5 different species of marine diatoms. After HRM analysis, we obtained different Tm values, and the results are summarized in Table 6. The results showed that the DNA sequences amplified by different primers were different. Moreover, the DNA sequences amplified by the same primer from different algae also varied. Therefore, the melting temperatures obtained after HRM analysis exhibited some differences.

**Table 6:** Tm value of diatoms with different primers ( °C).

Primer	*Navicula sp.*	*Odontella sp.*	*Skeletonema sp.*	*Chaetoceros sp.*	*Nitzschia sp.*
*UPA99*	79.03	78.37	79.27	79.17	79.17
*18S-V7*	81.35	82.40	82.85	81.30	82.03
*ND*	76.80	76.42	76.50	77.50	76.57
*Primer1*	83.40	84.72	82.43	84.42	84.08

### The result of sequencing the target sequence

After analyzing the PCR products using HRM, we went on to perform Sanger sequencing. By analyzing the sequencing peak charts, we obtained the DNA sequences of different diatom samples amplified with different primers (Table 7), which correspond to the target fragment sequences of the primers used in this study.

**Table 7:** DNA sequences obtained by amplifying different diatoms with different primers.

Primer	Species	DNA sequences
*UPA99-F*/ *UPA99-R*	*Navicula sp.*	CCTCTTATGCTTTACACTCTAGATACGATTTCTACCCGTTCAGAGGAAACTTTGTACGTTTCC
	*Odontella sp.*	CCTTTATGCCTTTATACTCTAGATACGATTTCTATCCGTTCAGAGGAAACCTTTGTACGTTTCC
	*Skeletonema sp.*	TTATGCCTTTCACTCTAGATACGATTTCTACCCGTTCAGAGGATACCTTTGTACGTTTCC
	*Chaetoceros sp.*	CCTTTATGCCTTTACACTCTAGATACGATTTCTACCCGTTCAGAGGAAACCTTTGTACGTTTCC
	*Nitzschia sp*	CTCTTATGCCTTTACACTCTAGATACGATTTCTACACGTTCAGAGGAAACCTTTGTACGTTTCC
*18S-V7F*/ *18S-V7R*	*Navicula sp.*	TGTTTTCCTTGTGAAGGCAGGCACTTCTTAGAGGGACAATTGGTGTTTAGCCAATGGAAGTGGGAGGCAATAACAGGTCTGTGATGCC
	*Odontella sp.*	CCGCGGATGATCTTTCATTGGCGAGAGCTTCTTAGAGGGACGACCGTCTACTAGACGGAGGAAGATAGGGGCAATAACAGGTCTGTGATGCC
	*Skeletonema sp.*	CGCGATGGCTTTTCATTGGCGAGGTCTTCTTAGAGGGACGTTCATTCTACAAGATGAAGGAAGATGGCGGCAATAACAGGTCTGTGATGCC
	*Chaetoceros sp.*	CGATGACTTTTCATTGGCGATGGTTTCTTAGAGGGACATGTAGTACAAAGCTACAGGAAGATTGGGGCAATAACAGGTCTGTGATGCC
	*Nitzschia sp*	GAGTGAGTTTTCACTGGGTGAAGCTTCTTAGAGGGACGTGCATTCTATTAGATGCAGGAAGATAGGGGCAATAACAGGTCTGTGATGCC
*ND-F*/*ND-R*	*Navicula sp.*	ATCGAGGTTTAAGGTGGTTTAGACTTCTTAAAAGATGATGAGAACATTAACTCTCAACCATTCATGCGTTGGAGAGAA
	*Odontella sp.*	AATGAGGTTTAAGGTGGTTTAGACTTCTTAAAAGATGATGAAAACATTAACTCTCAACCATTCATGCGTTGGAGAGAA
	*Skeletonema sp.*	ATGACGTTTAAAGGTGGTTTAGACTTCTTAAAGGATGATGAAAATATTAACTCTCAACCATTCATGCGTTGGAGAGAA
	*Chaetoceros sp.*	TTTGAGTTTAAAGGTGGTTTAGACTTCTTAAAAGACGATGAGAACATCAATTCTCAACCATTCATGCGTTGGAGAGAA
	*Nitzschia sp*	ATGAGGTTTAAAGGTGGTTTAGACTTCTTAAAAGATGATGAAAACATTAACTCTCAACCATTCATGCGTTGGAGAGAA
*Primer1-F*/ *Primer1-R*	*Navicula sp.*	TATTCAGCTCATAGCGTATATTAAAGTTGTTGCAGTTAAAAAGCTCGTAGTTGGATTTGTGGCATTGGTTGTGGCGTCCATTGATTTGGTTTTGCTGCGATCGTGCCATCCTTGGGTGGATTTTGTGTGGCATTAGGTTGTCGTGCAGG
	*Odontella sp.*	TATTCAGCTCATAGCGTATATTAAAGTTGTTGCAGTTAAAAAGCTCGTAGTTGGATATCTGGTGGGAGCAATGGGTCTCGCGCTTAGCGCGGGTACTTCAGTTGACTCCGGCCATCCTCGGGGAGAGCCCGTCTGGCATTAGGTTGTCGTGCAGG
	*Skeletonema sp.*	TCAGCTCATAGCGTATATTAAAGTTGTTGCAGTTAAAAAGCTCGTAGTTGGATTTCTGGCAGGAGTGACTGACCACAAACTCTGTTTGTGAGTTGTGTCATTCTGGCCATCCTTGGTAAGATCCTTTTTGGCATTAGGTTGTCGTGCAGG
	*Chaetoceros sp.*	TATTCAGCTCATAGCGTATATTAAAGTTGTTGCAGTTAAAAAGCTCGTAGTTGGATTTGTGGTGCGACGGATCGGTCCGACCTTTGGTGGGTACTCGATGTTGTCGCGCCATCCTTGAGTGGTTCGTCCTGGCATTAGGTTGTCGTGCAGG
	*Nitzschia sp*	TATTCAGCTCATAGCGTATATTAAAGTTGTTGCAGTTAAAAAGCTCGTAGTTGGATTTGTGGCTGTCGATAGCGGCCTGTCACTTAGTGTCAGTGTTTGCTGTCGTCGCCATCCTTGGGTGGAGCCTGTGTGGCATTAGGTTGTCGTGCAGG
*psaA-2-F*/ *psaA-2-R*	*Navicula sp.*	CGGTCCAGCTGTTATAAATCAGTCCATACAACTGTCCATGTTGCTGTAGAAGATTCACCTGCTACTGCTGCAGCAGCTTCTACTGGATCTACACCTGGTTGTGGTGTGATACGGA
	*Odontella sp.*	CGCTCCAGCTGTTATAAATCTGTCCAAACAACAGTCCAAGTAGCTGTTGAAGATTCACCAGCTACTGCAGCTGCAGCTTCTACTGGATCTACACCTGGTTGTGGTGTGATACGGA
	*Skeletonema sp.*	CGCTCCAGCTGTTATAAATCTGTCCATACAACAGTCCAAGTTGCAGTTGAAGATTCACCTGCTACAGCAGCAGCAGCTTCTACTGGATCTACACCTGGTTGTGGTGTGATACGGA
	*Chaetoceros sp.*	CGTTCCACGCTGTTATAAATCTGTCCAAACAACAGTCCATGTTGCTGTTGAAGATTCACCTGCTACAGCAGCGGCAGCTTCTACTGGATCAACACCTGGTTGTGGTGTGATACGGA
	*Nitzschia sp*	CGGTCCAGCTGTTATAAATCAGTCCATACAACTGTCCAAGTTGCTGTAGAAGATTCACCAGCTACTGCTGCTGCAGCTTCTACTGGATCTACACCTGGTTGTGGTGTGATACGGA
*Dia18S-F*/*Dia18S-R*	*Navicula sp.*	TGTGGCGTCCATTGATTTGGTTTTGCTGCGATCGTGCCATCCTTGGGTGGATTTTGTGTGGCATTAGGTTGTCGTGCAGGGGATGCCCATCCTTTACA
	*Skeletonema sp.*	ACTGACCACAACTCTGTTTGTGAGTTGTGTCATTCTGGCCATCCTTGGTAAGATCCTTTTTGGCATTAAGTTGTCGGGAAGGGGATGCCCATCCTTTACA
	*Nitzschia sp*	GTCGATAGCGGCTGTCACTTAGTGTCAGTGTTTGCTGTCGTCGCCATCCTTGGGTGGAGCCTGTGTGGCATTAGGTTGTCGTGCAGGGGATGCCCATCCTTTACA

### Calculation results of ΔH°, ΔS° and other parameters during prediction model building process

Based on the obtained DNA sequence, the corresponding GC content and number of base pairs were calculated. The GC content of all sequences was between 34.62% and 55.24%. The sequence lengths ranged between 60 bp and 152 bp, all of which were below 200 bp. Based on the four base arrangements of the DNA sequence and combined with the thermodynamic parameters of the nearest neighbor method, the total ΔH° and total ΔS° of each DNA sequence were calculated (Table 8).

**Table 8:** Parameters of different diatom DNA sequences with different primers.

Primer	Species	GC content	number of base pairs	ΔH° (kcal/mol)	ΔS° (cal/k·mol)
*UPA99-F*/ *UPA99-R*	*Navicula sp.*	42.19%	63	-510.4	-1390
	*Odontella sp.*	39.01%	63	-506.8	-1385.8
	*Skeletonema sp.*	41.67%	59	-467.8	-1272.8
	*Chaetoceros sp.*	42.19%	63	-510.3	-1388.9
	*Nitzschia sp.*	40.63%	63	-511.3	-1395.3
*18S-V7F*/ *18S-V7R*	*Navicula sp.*	47.73%	87	-706.9	-1898.3
	*Odontella sp.*	53.26%	91	-750.4	-2010.6
	*Skeletonema sp.*	49.45%	90	-743.7	-1997.9
	*Chaetoceros sp.*	45.45%	87	-708.3	-1912.9
	*Nitzschia sp.*	47.19%	88	-715.2	-1914.6
*ND-F*/ *ND-R*	*Navicula sp.*	37.18%	77	-617	-1681.8
	*Odontella sp.*	34.62%	77	-614.4	-1678.5
	*Skeletonema sp.*	34.62%	77	-615.2	-1681.5
	*Chaetoceros sp.*	35.90%	77	-619	-1688.5
	*Nitzschia sp.*	34.62%	77	-614.4	-1678.5
*Primer1-F*/ *Primer1-R*	*Navicula sp.*	44.30%	148	-1219.8	-3283.5
	*Odontella sp.*	51.61%	154	-1275.6	-3283.5
	*Skeletonema sp.*	44.00%	149	-1216.3	-3283.1
	*Chaetoceros sp.*	49.01%	150	-1237.2	-3318.3
	*Nitzschia sp.*	48.03%	151	-1232.8	-3318.3
*psaA-2-F*/ *psaA-2-R*	*Navicula sp.*	47.83%	114	-932	-2507
	*Odontella sp.*	47.83%	114	-934.6	-2512.9
	*Skeletonema sp.*	47.83%	114	-934.6	-2512.8
	*Chaetoceros sp.*	48.28%	115	-949.6	-2549.2
	*Nitzschia sp.*	47.83%	114	-932	-2507.1
*Dia18S-F*/ *Dia18S-R*	*Navicula sp.*	51.02%	97	-800.7	-2135.7
	*Skeletonema sp.*	46.00%	99	-800.9	-2153
	*Nitzschia sp.*	55.24%	104	-865.2	-2303.2

### Establishment results of the Tm value prediction model

We analyzed the total enthalpy and entropy values, GC content, number of base pairs, and the actual measured Tm values of 20 DNA sequences amplified by the four primer pairs: *UPA99-F*/*UPA99-R*, *18S-V7F*/*18S-V7R*, *ND-F*/ND-R, and *Primer1-F*/*Primer1-R*. From this, we came up with the following formula for predicting Tm values:

When the GC content is 40%≤GC content≤60%:


$$\begin{array}{c} Tm=\frac{\text{ΔH}}{\text{ΔS}}－0.27GC\%-\frac{150+2\text{n}}{\text{n}}－273.15 \end{array}$$

When the GC content is <40%:


$$\begin{array}{c} Tm=\frac{\text{ΔH}}{\text{ΔS}}-\frac{\text{GC\%}}{3}-\frac{150+2\text{n}}{\text{n}}－273.15 \end{array}$$

Among them, ΔH is the total enthalpy change of the sequence, ΔS is the total entropy change of the sequence, GC% is the GC content of the sequence, and n is the number of base pairs (one less than the sequence length).

### Verification of Tm value prediction formula

In order to verify the obtained formula, we used two pairs of primers: *psaA-2-F*/*psaA-2-R* and *Dia18S-F*/*Dia18S-R*, measuring the total enthalpy, total entropy, GC content, and number of base pairs for eight DNA sequences. We calculated the predicted Tm values for these sequences (Table 9). At the same time, we compared the predicted values with the measured ones. The comparison analysis shows that the average error of the predicted Tm values is within 1 °C. This result indicates that the formula we developed can predict Tm values quite accurately and can be effectively used to identify the species of unknown samples.

**Table 9:** The measured and predicted Tm values of two pairs of primers (°C).

Species	*PsaA-2*			*Dia18S*		
	Predicted values	Measured values	Difference	Predicted values	Measured values	Difference
*Navicula sp.*	81.68	82.38	0.7	83.3	84.43	1.13
*Odontella sp.*	82.05	82.54	0.49	–	–	–
*Skeletonema sp.*	82.2	82.56	0.36	81.5	82.91	1.41
*Chaetoceros sp.*	82.78	83.05	0.27	–	–	–
*Nitzschia sp.*	81.97	82.34	0.37	84.75	84.14	0.61

**The average difference is calculated to be 0.67** °C

**Note: “-” means that the primer cannot detect this species, so it has no detection value.**

## Discussion

The few current studies on Tm value prediction methods only started half a century ago [23,24]. These prediction methods can be roughly divided into two categories. One is the Tm value prediction method based on nucleotide sequence composition, and the other is the Tm value prediction method based on the nearest neighbor method. Their prediction accuracy varies. At present, the latest Tm value prediction technology that we can access is the Tm value prediction salinity correction formula proposed by Owczarzy et al [25]. This method performs salinity correction on the basis of the nearest neighbor prediction method to improve its prediction accuracy. Although this salinity correction method is more accurate than previously proposed algorithms, the formula is based on melting experiments performed on 92 DNA duplexes with lengths ranging from 10 to 30 bp. Therefore, this formula can only accurately predict the melting temperature of 10–30 bp DNA double strands. Obviously, it cannot meet the needs of actual HRM application fields. Since the length of most PCR amplification products used for HRM analysis usually does not exceed 300 bp [2,26]. We use DNA sequences with a length of about 50–200 bp for Tm value prediction. This makes the method highly applicable and versatile in practical applications. The implementation of the previous method required precise determination of parameters such as the molar concentration of DNA molecules and the concentration of sodium ions in the reaction system. Accurate measurement of these parameters is difficult to implement in practice, thus limiting the widespread application of these methods. The method we proposed only needs to calculate the total enthalpy value, total entropy value, GC content, number of base pairs, and other parameters of the DNA sequence. These are easy to obtain and are ideal for use in grassroots laboratories. Therefore, it is extremely necessary to explore simple, fast, and accurate prediction methods.

When DNA double strands are unzipped into single strands, it is necessary to destroy the stacking effect between adjacent bases and the hydrogen bonds between complementary paired bases [24,27]. To destroy them, sufficient thermal energy needs to be provided, which is why the melting temperature is formed. Therefore, Crothers et al.(1964) took the lead in proposing to use the nearest neighbor method model for the study of nucleic acid thermodynamics. This model converts the energy calculation of the mutual transformation process of DNA double-stranded and single-stranded DNA into the sum of the standard enthalpy change and entropy change of the dimer formed by the four bases of the DNA molecule. Compared with methods that only consider nucleotide sequence composition, the nearest neighbor model can better reflect the interaction of adjacent base pairs, which also increases the prediction accuracy of the model. At the same time, the model has good adaptability and can be used for different DNA sequences, regardless of length. By simply replacing the corresponding base pairs, the corresponding enthalpy change and entropy change results can be quickly obtained. Although the nearest neighbor method model also has its shortcomings, due to its good theoretical foundation, this model has been used more frequently in recent years. The nearest neighbor model mainly considers the order of base pairs in the DNA double strand. However, the factors that affect the Tm value are not only the order of the four bases in the nucleotide chain but also the length of the nucleotide chain and the content of each base, especially the GC [28]. Therefore, by combining the nearest neighbor method model, we used four pairs of primers *UPA99-F*/*UPA99-R*, *18S-V7F*/*18S-V7R*, *ND-F*/ND-R and *Primer1-F*/*Primer1-R* to amplify 20 DNA sequence. Calculate the total enthalpy value, total entropy value, GC content, number of base pairs, and other parameters of these sequences, and then derive a new empirical formula to predict the Tm value.

In this study, we used seawater diatoms as the experimental subjects. Its DNA sequence is obtained through specific amplification, and then HRM is used to determine the true Tm value. Based on these measured data, we further calculated and derived the empirical formula for Tm value prediction. This process ensures that the prediction model established can closely match the actual application scenario. At the same time, we used 8 DNA sequences amplified by two pairs of primers *psaA-2-F*/*psaA-2-R* and *Dia18S-F*/*Dia18S-R* to verify the method. Compared with the measured Tm value, the average error range is within 1 °C. Therefore, this method has high prediction accuracy and practicality. However, since the sample size used by this prediction method is not large enough, a large number of experimental samples are needed for further verification. In addition, the DNA sequence length we currently use to predict Tm values is around 50–200 bp, but this formula cannot reflect the prediction effect of DNA sequences above 300 bp. In the future, we will further verify and adjust the formula for DNA sequences longer than 300 bp. It can be seen from the experimental results that the measured Tm values of *Chaetoceros sp.* and *Nitzschia* *sp.* amplified by primers *UPA99-F*/*UPA99-R* are the same. This may be due to the similar DNA sequence composition and close Tm values of the two algae. However, due to temperature fluctuations between holes, the actual measured Tm values are the same. This situation can be eliminated through multiple experiments and does not affect the prediction of Tm values in this study. At the same time, it is precisely because of the temperature difference between the holes that the sensor readings are uneven, which affects the accuracy of the measurement, which further illustrates the importance and necessity of our Tm value prediction.

In this study, the template DNA was first amplified by PCR, and then the obtained PCR product was directly analyzed by HRM. When we change the Mg^2+^ concentration in the reaction system, there are two discussions(S1~2 Tables):

(1) The Mg^2+^ concentration in the PCR reaction system is a key factor affecting the PCR amplification efficiency, so it indirectly affects the concentration of the PCR product. Through our research, we found that the influence of PCR product concentration on the Tm value is within the permissible error range.
(2) Each component in the PCR reaction system has its own optimal concentration range. In this experiment, we chose to increase the concentration of Mg^2+^ to investigate the effect of ion concentration on the Tm value. Through the experiment, we obtained the Tm values of the PCR products at different Mg^2+^ concentrations. Analysis of the obtained results reveals that an increase in Mg^2+^ concentration exerts a discernible influence on the Tm value. Although the rate of increase in the Tm value gradually decelerates with rising concentration, a significant augmentation in the average error is observed when the Mg^2+^ concentration surpasses 2mM(mmol/L) (with the average error reaching 1.77). Within the PCR reaction system, the concentrations of other ions remain relatively stable, with only the Mg^2+^ concentration varying within the standard range (1.5 mM to 3.5 mM). Therefore, when employing this method for Tm value detection, it is imperative to regulate the Mg^2+^ concentration in the PCR system to within the range of 1.5 mM to 2 mM.

This study established a method for predicting Tm values in HRM analysis using marine diatoms as experimental subjects. Given the basic principles of HRM technology, this formula should theoretically apply to Tm prediction in all HRM research systems that meet the conditions. It requires further refinement and correction through application and research practice in related fields. However, the application of this method is not limited to determining the species of diatoms to solve forensic problems, but also has broad interdisciplinary applicability, such as forensic entomology [29], herbal medicine [30], wildlife [31], and the identification of bacteria [32], viruses [33], and other microorganisms. These related disciplines can use this method to solve practical problems encountered. In summary, this Tm value prediction method provides new methods and ideas for solving practical problems in multiple related fields.

## Conclusions

In summary, in this study, we took marine diatoms as the research object, conducted melting experiments on PCR products, and used the nearest neighbor method model. Starting from actual species identification experiments, we initially established a method for predicting Tm values in HRM analysis. At the same time, the average error in predicting Tm values by this method is within 1 °C, indicating that it is simple, efficient, has high prediction accuracy, practicality, and broad interdisciplinary applicability. By establishing this method, species can be identified more effectively, especially to facilitate species identification of large biological populations in marine ecosystems. Although certain results have been achieved in the study of methods for predicting Tm values, there are still some limitations. Specifically, the applicability of this method is limited by the experimentally verified range of Mg^2+^ concentration. The Mg^2+^ concentration is preferably between 1.5mM to 3.5 mM. In future research, we can obtain a large amount of experimental data and combine it with machine learning algorithms to deeply explore the characteristics and patterns to make the prediction of Tm values more accurate and convenient. At the same time, we can explore new theoretical models for DNA melting to provide a better theoretical basis for predicting the Tm value of the melting curve.

## Supporting information

S1 Table Tm values obtained from HRM analysis of PCR products with different DNA concentrations (°C). Amplification of four diatoms using primers psaA-2-F/psaA-2-R, with different DNA template concentrations and amplification cycle numbers. C_DNA_ is the concentration of template DNA.
(DOCX)

S2 Table Tm values obtained from HRM analysis of PCR products under different Mg^2+^ concentrations (°C). Different concentrations of Mg²⁺ are added in different PCR systems.
(DOCX)
