# Supplementary material for: Establishment of a simple prediction method for DNA melting temperature: high-resolution melting curve analysis of PCR products
Source: PLoS One. 2025 Apr 16;20(4):e0321885. doi: 10.1371/journal.pone.0321885 (PMC12002481; doi:10.1371/journal.pone.0321885)
Supplement: S1 Table — Amplification of four diatoms using primers psaA-2-F/psaA-2-R, with different DNA template concentrations and amplification cycle numbers. CDNA is the concentration of template DNA. (DOCX) [file pone.0321885.s001.docx]

**S1 Table. Tm values obtained from HRM analysis of PCR products with different DNA concentrations (℃)**

| Species | C_DNA_（ng/μl） | Cycles | | |
| --- | --- | --- | --- | --- |
|  |  | 30 | 35 | 40 |
| *Navicula sp.* | 0.5 | 81.83 | 81.93 | 81.90 |
|  | 1 | 81.90 | 81.85 | 81.92 |
|  | 1.5 | 81.82 | 81.95 | 81.98 |
| *Odontella sp.* | 0.5 | 81.98 | 82.03 | 82.08 |
|  | 1 | 81.82 | 82.00 | 82.00 |
|  | 1.5 | 82.07 | 81.85 | 82.35 |
| *Skeletonema sp.* | 0.5 | 82.22 | 82.15 | 82.45 |
|  | 1 | 82.05 | 82.15 | 82.38 |
|  | 1.5 | 82.27 | 82.30 | 82.62 |
| *Nitzschia sp.* | 0.5 | 81.98 | 81.95 | 81.95 |
|  | 1 | 81.85 | 82.00 | 82.15 |
|  | 1.5 | 81.90 | 81.88 | 82.47 |

**Amplification of four diatoms using primers *psaA-2-F*/*psaA-2-R*, with different DNA template concentrations and amplification cycle numbers. C_DNA_ is the concentration of template DNA**
