# Supplementary material for: Establishment of a simple prediction method for DNA melting temperature: high-resolution melting curve analysis of PCR products
Source: PLoS One. 2025 Apr 16;20(4):e0321885. doi: 10.1371/journal.pone.0321885 (PMC12002481; doi:10.1371/journal.pone.0321885)
Supplement: S2 Table — Different concentrations of Mg²⁺ are added in different PCR systems. (DOCX) [file pone.0321885.s002.docx]

| Primers | Species | Mg^2+^（mmol/L） | | | | |
| --- | --- | --- | --- | --- | --- | --- |
|  |  | 1.5 | 2 | 3 | 4 | 5 |
| *psaA-2-F*/  *psaA-2-R* | *Navicula sp.* | 82.27 | 82.72 | 83.45 | 83.65 | 83.85 |
|  | *Skeletonema sp.* | 83.45 | 83.67 | 84.08 | 84.28 | 84.30 |
|  | *Nitzschia sp.* | 82.57 | 83.02 | 83.65 | 83.88 | 83.42 |
| *Primer1-F*/  *Primer1-R* | *Navicula sp.* | 84.05 | 84.50 | 85.25 | 85.53 | 85.70 |
|  | *Skeletonema sp.* | 83.05 | 83.72 | 84.30 | 84.58 | 84.82 |
|  | *Nitzschia sp.* | 84.88 | 85.55 | 86.00 | 86.30 | 86.43 |

**S2 Table. Tm values obtained from HRM analysis of PCR products under different Mg^2+^concentrations (℃)**

**Different concentrations of Mg²⁺ are added in different PCR systems.**
